# The U.S. Health Care System on the Eve of the Covid-19 Epidemic: A Summary of Recent Evidence on Its Impaired Performance

**DOI:** 10.1177/0020731420937631

**Published:** 2020-06-30

**Authors:** David U. Himmelstein, Steffie Woolhandler

**Affiliations:** 1 City University of New York at Hunter College, New York, USA; 2 Harvard Medical School, Cambridge, MA, USA

**Keywords:** health care, United States, COVID-19, neoliberalism, health inequalities

## Abstract

Four decades of neoliberal health policies have left the United States with a health care system that prioritizes the profits of large corporate actors, denies needed care to tens of millions, is extraordinarily fragmented and inefficient, and was ill prepared to address the COVID-19 pandemic. The payment system has long rewarded hospitals for providing elective surgical procedures to well-insured patients while penalizing those providing the most essential and urgent services, causing hospital revenues to plummet as elective procedures were cancelled during the pandemic. Before the recession caused by the pandemic, tens of millions of Americans were unable to afford care, compromising their physical and financial health; deep-pocketed corporate interests were increasingly dominating the hospital industry and taking over physicians’ practices; and insurers’ profits hit record levels. Meanwhile, yawning class-based and racial inequities in care and health outcomes remain and have even widened. Recent data highlight the failure of policy strategies based on market models and the need to shift to a nonprofit social insurance model.

As we go to press, many small medical practices and clinics are on the brink of bankruptcy, and reports of massive layoffs and furloughs by major nonprofit hospitals and health systems have begun to appear. As of May 13, 255 U.S. hospitals have announced layoffs, furloughs, or reduced hours and wages for staff.^1^ These cuts have affected, for example, 2,800 workers at Detroit’s Henry Ford Health System, 4,100 at Cleveland’s University Hospital system, and 30,000 at the Mayo Clinic. Other health systems have cut workers’ wages – for example, Stanford Health Care in Palo Alto, which reduced the pay of doctors, nurses, and other workers by 20%.^2,3^ Steward, one of the nation’s largest for-profit hospital system, cut physicians’ salaries by 20% and is furloughing workers, including some who were providing coronavirus testing. The hospital firm is owned by Cerberus Capital, where former U.S. Vice President Daniel Quayle holds the title of Chairman for Global Investment. The company said it was experiencing a “seismic financial shock” because “Elective surgeries are the cornerstone of our hospital system’s operating model – and the negative impact due to the cancellations of these procedures cannot be overstated.”^4^

Between March and April of this year, the ambulatory care workforce fell by 1.188 million persons, a 14.9% drop, while the number of hospital employees decreased by 135,000, a fall of 2.6%. Meanwhile, the number of insurance company employees fell by less than 0.2%.^5^

## Access to Care and Medical Costs

A randomized trial carried out by the U.S. Treasury Department has again confirmed that health insurance saves lives. In 2017, the Internal Revenue Service sent informational letters to 3.9 million taxpayers randomly selected from the 4.5 million who had paid tax penalties for failing to comply with the coverage mandate of the Affordable Care Act (ACA). The letters provided information about penalties and insurance plan costs, together with instructions on how to investigate the availability of Medicaid and ACA exchange coverage. In the subsequent 2 years, coverage rates were 1.3 percentage points higher among persons who received a letter. Moreover, the death rate among adults age 45 to 65 who received a letter (vs those who did not) was 0.06 percentage points lower, equivalent to 1 death averted for every 1,648 individuals in that age group who were sent a letter.^6^

When Americans turn 65 and gain Medicare coverage, cancer detection rates, particularly for early-stage disease, increase sharply for breast, colorectal, and lung cancer, and mortality rates fall.^7^

Yet another indicator that Americans are struggling to afford care: About 8 million people have started a crowdfunding campaign to pay for medical care for themselves or a household member on sites such as GoFundMe. Another 12 million people started campaigns for someone outside of their household.^8^

A new study of the burden of health costs borne by different income groups – the first to encompass costs for institutionalized persons – suggests that the U.S. health care financing system is even more regressive than previous work had indicated. Total health care payments, including premiums paid by workers and their employers, taxes paid, and out-of-pocket spending, consumed 33.9% of income for the poorest fifth of the population, but only 16% for the wealthiest fifth.^9^

A new NPR/Robert Wood Johnson Foundation/Harvard School of Public Health survey has found that even wealthy Americans sometimes have difficulty affording care. Among the wealthiest 1% of adults (those with household incomes above $500,000), 5% reported a serious problem paying medical bills within the past few years, 5% had a serious problem paying for prescription drugs, and 7% reported a serious problem getting health care when needed. Among the wealthy, 10% said they had failed to fill a prescription or had cut back on dosages in the past year due to prescription costs. Lower-income adults encountered all of the cost and access problems much more frequently.^10^

In the decade ending in 2018, the average premium for an employer-sponsored family plan increased from $12,298 to $19,565; the employee’s share of the premium increased from $3,394 to $5,431; and the average deductible increased from $1,445 to $2,992. Meanwhile, median household income increased modestly, from $53,000 to $64,202 (not adjusted for inflation).^11^

By eliminating the financial burden of copayments, deductibles, and co-insurance, Improved Medicare for All would cut the poverty rate by 18.8%, according to data from the U.S. Census Bureau’s March 2019 Current Population Survey. Out of the 41.5 million people living below the poverty line, cutting cost-sharing for health care would lift 8 million out of poverty.^12^

An official estimate from the Centers for Medicare and Medicaid Services (CMS), released prior to the COVID-19 epidemic, predicted that U.S. health expenditures would grow by 5.4% annually between 2019 and 2028, reaching $6.2 trillion (19.7% of gross domestic product) by the end of the period.^13^

## The Harms of Copayments

Providing essential medications without charge increased adherence to treatment and significantly lowered blood pressure among Canadians prescribed an antihypertensive drug, according to a randomized, controlled trial in Ontario. (Canada’s single-payer system does not include universal drug coverage, an omission that the ruling Liberal Party promised to address during the election campaign last fall.) Hemoglobin A_1c_ and LDL cholesterol levels also fell slightly, but non-significantly, in the free medications group.^14^

A new study finds that Veterans Health Administration (VA) patients, relative to Americans with non-VA coverage, are only about half as likely to skip a prescribed medication because of costs (6.1% vs 10.9% of others), despite VA patients having lower average incomes. VA coverage especially improved drug adherence among people with chronic illnesses, while shrinking racial/ethnic and income-related disparities. The VA provides free prescription drugs to some patients, while charging others copays of $5 per month for preferred generics and $11 per month for brand-name drugs, with annual out-of-pocket drug costs capped at $700. Yet despite charging patients less, the VA’s drug spending is lower than that of private insurers because it pays drug manufacturers lower prices.^15^

When the Dutch government instituted new copayments for mental health care, the use of mental health services for both severe and mild disorders decreased abruptly and persistently, particularly in low-income neighborhoods. Meanwhile, the number of involuntary commitments increased by 96.8% and episodes of acute mental health care rose by 25.1%. While overall costs decreased by $15.1 million, costs increased $28.8 million for adults with psychotic or bipolar disorders.^16^

## Medicare’s Flaws

A recent study indicates why the US advocacy organization Physicians for a National Health Program calls for *Improved* Medicare for All. Among seriously ill Medicare enrollees (i.e., those who have visited 3 or more physicians and been hospitalized at least twice in the past year), 53% had a serious problem paying a medical bill, 36% had used up all or most of their savings, 27% had been contacted by a collection agency, and 23% were unable to pay for basic necessities.^17^

While almost all persons older than 64 are covered by Medicare, 65% of them lack dental coverage, and each year about 10% of them skip needed dental care because they cannot afford it. About 20% of seniors have lost all of their teeth, and many more have tooth and gum problems that compromise their nutritional status and pose other health threats.^18^

Medicare provides full coverage for the first 20 days of skilled nursing facility care, but after that, patients are responsible for copayments of more than $150 (all dollar amounts in U.S. dollars) per day. Skilled nursing facility discharge rates for Medicare patients spike on the 20th day of their stay and are especially high for black and Hispanic patients and those from low-income ZIP codes. Patients discharged on Day 20 also had significantly more comorbidities than those discharged on other days, indicating that financial rather than medical reasons motivated their discharges.^19^

## Race and Mortality

Recent declines in U.S. life expectancy and deaths due to “diseases of despair” among working-age white people have gained wide attention. But a new study highlights rising mortality rates among working-age people of color and other disturbing trends dating to the 1990s and earlier. Population-wide life expectancy improved dramatically between 1959 and the early 1980s, but improvement slowed starting in the 1980s, plateaued starting in 2011, and deteriorated between 2014 and 2017. Midlife mortality has been increasing since 2010, and a wide variety of causes – not just diseases of despair – have contributed to the increase. Native Americans have suffered the worst midlife mortality increases (a 34% increase since 2000), and they now have the highest death rates of any race/ethnicity group (59% higher than non-Hispanic whites). Midlife mortality for blacks began increasing in 2014 and is currently 49% higher than non-Hispanic whites. Although each of these groups experienced large increases in fatal drug overdoses (with the largest increase – 172% since 2010 – among the black population), deaths from many other causes also increased.^20^

Maternal mortality was 252% higher for blacks than for non-Hispanic whites in 2018 (37.1 vs 14.7 per 100,000 live births), according to new data from the Centers for Disease Control and Prevention. Hispanics’ maternal mortality rate was the lowest, at 11.8 per 100,000.^21^

## Socioeconomic Inequality

Many wealthy nations have poverty rates comparable to the United States’ before accounting for the effect of social programs. But their more robust safety nets cut their poverty rates to levels far lower than ours. While government programs cut the poverty rate in the United States from 26.6% to 17.8%, comparable figures for Finland are 34.2% to 5.8%; for Denmark, 24.6% to 5.8%; for France, 37% to 8.3%; for Germany, 32.7% to 10.4%; and for Italy, 33.8% to 13.7%.^22^

In the United States, the ratio of chief executive officers’ (CEOs’) pay to the average workers’ income rose from less than 16:1 in 1965 to 221:1 in 2018.^23^

## Insurers, Billing, and Paying

Between 2008 and 2018, private insurance costs per enrollee grew by 52.6%, far faster than cost growth in Medicare (21.5%) or Medicaid (12.5%).^24^

For big health insurers, 2019 was a banner year. The profits of the 7 largest U.S. publicly traded insurers increased 66% from 2018, to $35.6 billion, driven by a wave of mergers and acquisitions.^25^

Hospitals and outpatient practices may be struggling, but UnitedHealth, the nation’s largest private insurance firm, is doing just fine. In the quarter ending March 31, 2020, the company made a profit of $5 billion, an increase of $164 million over the same quarter in 2019. Its “medical loss ratio,” the portion of premiums that actually pay for care, fell to 81% (implying an overhead of 19%) from 82% in 2019.^26^

Yet another Medicare Advantage (MA) scam: Collect premiums from Medicare, while patients get their care from the VA. About 1.2 million veterans are dually enrolled in an MA plan and the VA. During a 3-year period, one quarter of dual MA/VA enrollees undergoing coronary revascularization procedures had their procedures at a VA facility, increasing costs for the VA system – and saving MA plans – $214.7 million.^27^

The Justice Department has filed suit against Anthem (owner of many for-profit Blue Cross plans) for Medicare fraud, charging that the giant insurer combed MA enrollees’ charts for additional diagnoses that would boost the MA premiums paid by CMS, but then failed to delete inaccurate diagnostic codes that it discovered.^28^ Anthem’s chart reviews generated about $100 million annually in extra payments between 2014 and 2018. The suit comes after a Department of Health and Human Services Inspector General’s report found that MA plans had increased their risk-adjusted payments by $6.7 billion in 2017 based on chart reviews, and that for 41% of the chart review-based diagnoses, no visits, procedures, tests, or supplies were recorded in the chart. Hence, according to the Inspector General, while “beneficiaries may not have received any other services for the … diagnoses … Medicare paid billions in MA risk-adjusted payments to provide care for these beneficiaries.”^29^

More than 1 in 5 (20.5%) privately insured patients undergoing surgery with an in-network surgeon and facility received a “surprise” bill for out-of-network care, most commonly for an out-of-network surgical assistant or anesthesiologist. The bills averaged $2,011; patients covered by ACA exchange plans more frequently received out-of-network bills (27% of procedures for ACA exchange plans vs 20% for non-exchange plans).^30^

Surprise bills are even more likely after an emergency department (ED) visit or overnight hospital stay: 42.8% of visits by privately insured patients to an in-network ED resulted in a “surprise” bill for out-of-network care in 2016, up from 32.3% in 2010. Similarly, 42.0% of privately insured inpatient admissions at in-network hospitals resulted in an out-of-network bill, up from 26.3% in 2010. In 2016, the out-of-network bills averaged $628 per ED visit and $2,040 per inpatient stay.^31^

## Corporate Medicine

The Partnership for America’s Healthcare Future, an “astroturf” group funded largely by insurance and drug firms to oppose reforms that threaten their interests, bought half of all political ads in Iowa during the runup to the presidential primary in the summer of 2019. Most of the ads bashed Medicare for All.^32^

Hospital leaders and policy wonks often claim that the recent wave of hospital mergers and acquisitions will improve efficiency and upgrade quality. However, previous studies found that the consolidation of hospital ownership has raised prices, and a new analysis refutes the claim that mergers improve quality. Performance on measures of patient experience declined at 246 hospitals acquired by a larger system between 2009 and 2013 as compared to other hospitals. Neither mortality rates nor hospital readmission rates improved in the newly acquired hospitals.^33^

Affiliation with a large system has also been trumpeted as a solution to the woes of struggling rural hospitals. But a new study indicates that, although affiliation improved rural hospitals’ finances, it led to significant reductions in the availability of diagnostic imaging technologies, obstetric and primary care services, and outpatient care.^34^

Many “nonprofit” health systems rake in enormous profits. In 2018, Kaiser Foundation Health Plan and Hospitals had an operating surplus of $1.9 billion. Other big gainers included: Mayo Clinic ($706 million), Indiana University Health ($612 million), Intermountain Health ($547 million), the University of Pennsylvania Health System ($383 million), New York-Presbyterian ($340 million), and Partners HealthCare System, now renamed Mass General Brigham ($310 million).^35^

Many leading academic medical centers are highly profitable, but provide only modest amounts of care for Medicaid or uninsured patients. The Mayo Clinic’s record is especially egregious, and its CEO openly instructed employees to prioritize privately insured patients over those covered by Medicaid. The Clinic realized an operating surplus of $706 million in 2018, but charity care accounted for only 0.66% of expenses (half the level of the Cleveland Clinic), and just 3% of medical care revenue came from Medicaid, versus 8% at Cleveland Clinic and 6.5% at Cedars Sinai in Los Angeles.^36^

Hospitals in New York have filed almost 31,000 lawsuits against patients since 2015 seeking to collect on unpaid bills. The suits included 2,749 by New York University’s Winthrop Hospital and 2,233 by Northwell’s North Shore University Hospital.^37^

Many EDs have closed in recent years, either because hospitals have been shuttered entirely or because hospitals that stayed open wanted to avoid often-unprofitable ED patients. A recent study found that when EDs closed and patients’ driving time to the nearest ED increased by 30 minutes or more, the proportion of heart attack patients who received a percutaneous coronary intervention fell, and both mortality and readmission rates increased.^38^

Kidney transplantation is the optimal treatment for most patients with end-stage renal disease (ESRD), but only 14% of U.S. patients newly diagnosed with ESRD receive a transplant or are placed on a transplant waiting list within 1 year. ESRD patients cared for at nonprofit dialysis facilities are 3 times more likely to be placed on a transplant waiting list and 60% more likely to actually receive a transplant than patients at for-profit facilities, most of which are owned by 2 giant firms, Fresenius and Davita.^39^

Dr. Michael Apkon has up-close experience with care on both sides of the U.S./Canada border. He was the chief medical officer at Children’s Hospital of Philadelphia before serving as the CEO of the Hospital for Sick Children in Toronto. He returned to the United States to become CEO of Tufts Medical Center in 2018. According to Dr. Apkon:

What I saw was the impact [Canada’s single-payer system] has on the ease of access, the quality of the outcomes, and the social justice of the system … The moral distress [due to] barriers that are there because of the way the system is constructed, those were issues that I never faced in Ontario … What I came to appreciate in Canada is that there are things that markets are just not built to do.^40^

## Pay-for-Performance, Accountable Care Organizations, and “Paying for Value, Not Volume”: More Evidence of Failure

“Next Generation” Accountable Care Organizations (ACOs) not only failed to save money, but actually increased Medicare’s costs by $93.9 million during 2016 and 2017, according to a University of Chicago study funded by CMS. Although the ACOs cut Medicare spending on care by $123.2 million, the bonuses CMS paid them greatly exceeded the savings. The findings add to the growing evidence that ACOs either increase costs or (at best) achieve only trivial savings.^41^

In California, 59% of the quality measures for Medicaid-managed care plans have been stagnant or deteriorated since 2008. Three of the 9 child-health measures currently in use have worsened, while 3 others remained stagnant. For-profit plans scored markedly worse than nonprofit or public plans.^42^

A new study refuted the assertions of a widely cited 2011 *New Yorker* article by Atul Gawande that a “hotspotting” program in Camden, New Jersey, markedly reduces health care costs and improves quality among “superutilizers,” patients with very high use of health services. The new study randomly assigned 800 patients to either the “hotspotting” intervention in Camden or a control group that received usual care. The utilization of care fell markedly over time in both the intervention and control groups, but there was no difference in readmissions, hospital days, or hospital costs over 180 days.^43^

Another randomized clinical trial found that a chronic disease management program for patients hospitalized with chronic obstructive pulmonary disease (COPD) actually worsened outcomes. The study compared usual care to an intervention that included transition support by specially trained nurses for 30 days after hospital discharge to assure adherence with discharge plans and connection to outpatient care, as well as 3 months of help for patients and families with self-management of COPD. But the intervention group had more hospital admission and ED visits than the control group, without any improvement in patients’ quality of life.^44^

## The Perils of Electronic Medical Records

Primary care doctors and non-surgical specialists spent on average more than 16 minutes per ambulatory encounter using electronic health records (EHRs), according to a study of about 100 million encounters with 155,000 physicians who used Cerner’s EHR. One third of EHR time was spent on chart review, about one quarter on documentation, and 17% on entering orders; 11% of the interactions occurred on nights and weekends. The authors estimate that using the EHR took about 5 minutes longer per encounter than using paper records.^45^
